# circITCH suppresses cell proliferation and metastasis through miR‐660/TFCP2 pathway in melanoma

**DOI:** 10.1002/cam4.4627

**Published:** 2022-03-10

**Authors:** Jianfei Zhang, Yanlin Cai, Shunliang Sheng, Congling Zhao, Bin Jiang

**Affiliations:** ^1^ Department of Plastic surgery, The Second Affiliated Hospital, Hengyang Medical School University of South China Hengyang Hunan China; ^2^ Department of Gynaecology and Obstetrics, The First Affiliated Hospital, Hengyang Medical School University of South China Hengyang Hunan China; ^3^ Department of Pediatrics Guangdong Women and Children Hospital Guangzhou China

**Keywords:** ceRNAs, circITCH, melanoma, miR‐660, TFCP2

## Abstract

**Background:**

Melanoma is an aggressive disease that is rising in incidence. Advanced melanoma is still a life‐threatening disease. CircRNAs are documented to be involved in melanoma progression. But circITCH role in melanoma remains unclear.

**Methods and Results:**

To explore the functions of circITCH in melanoma, levels of circITCH in melanoma tissues and paracarcinoma normal tissues were detected. To study the roles of circITCH in melanoma in terms of cell proliferation and migration, in vitro and in vivo experiments were performed. Mechanism study was designed to investigate the potential regulatory effect of circITCH in melanoma. Results revealed that circITCH expression was repressed in melanoma versus adjacent normal tissues. Function study showed that circITCH suppressed melanoma cell proliferation and metastasis. The mechanism study showed that circITCH‐sponged miR‐660 to upregulate TFCP2 and suppress melanoma progression.

**Conclusions:**

The circITCH/miR‐660/TFCP2 axis is involved in melanoma progression hence circITCH can be a diagnostic biomarker as well as a target for treating melanoma.

## 1 INTRODUCTION

Skin malignant melanoma is a tumor produced by melanocytes of the skin and other organs.^1^ The pathogenesis of skin malignant melanoma still remains unknown. A global data analysis showed that the most important risk factor for the disease is excessive exposure to ultraviolet light.^2^ At present, WHO has designated indoor sunbathing as a carcinogen.^3^ Data from the United States in 2018 indicate that age, race, and gender are also independent risk factors for malignant melanoma.^4^ Although adjuvant immunotherapy or targeted therapy have extended survival, melanoma is still deadly owed to poor prognosis and premature metastasis. For patients diagnosed with stage IV melanoma, 5‐year survival is only 19%.^5^ The survival rate of early‐stage patients is much higher than that of late‐stage patients. Thus, effective timely diagnosis and innovative therapies are essential and needed.

Circular RNA (circRNA) is a noncoding RNA (ncRNA) characterized by a covalent closed‐loop structure widely expressed in eukaryotic and prokaryotic cells. It lacks 5′ and 3′ free ends. It is not easy to be degraded by exonuclease R. In addition, most circRNAs are highly conserved among different species.^6^ circRNA is a large class of ncRNA with regulatory functions. circRNA mainly performs the following functions: as an miRNA sponge, posttranscriptional regulation, encoding protein, generating pseudogenes derived from circRNA, and affecting variable splicing.^7^ circRNA mainly acts as an miRNA sponge in skin‐related diseases. For example, hsa_circ_0084043 promotes the expression of Snail by sponge adsorption of miR‐153‐3p to increase the malignancy of melanoma.^8^ In arsenite‐treated human immortalized epidermal cells, hsa_circ_100284 acts as an miR‐217 sponge, upregulating the expression of its target gene EZH2, leading to acceleration of the cell cycle and causing malignant transformation.^9^

Circular RNA ITCH (circITCH), a tumor‐suppressive circRNA, is involved in multiple cancers.^10^ circITCH is an miRNA sponge of miR‐330‐5p and upregulates SIRT6, Survivin, and SERCA2a to ameliorate doxorubicin‐induced cardiotoxicity.^11^ In bladder cancer, circITCH stimulated the manifestation of p21 and PTEN via miR‐17 and miR‐224 sponging.^12^ In hepatocellular carcinoma, circITCH could sponge miR‐224‐5p and modulate the expression of MafF.^13^ However, the role of circITCH in melanoma remains indefinite.

Herein, we investigated the role of circITCH in melanoma. We demonstrated that circITCH was downregulated in melanoma tissues versus neighboring normal tissues. Several in vitro and in vivo studies revealed that circITCH suppressed melanoma cell proliferation and metastasis. Mechanism study demonstrated that circITCH‐sponged miR‐660 to upregulate TFCP2 and suppress melanoma progression. Summarily, this research disclosed the circITCH/miR‐660/TFCP2 axis role in melanoma and circITCH represented a novel diagnostic biomarker and potential targets for treating melanoma.

## 2 MATERIALS AND METHODS

### 2.1 Specimen collection

Fresh primary melanoma tissues and surrounding normal tissues were acquired at the Second Affiliated Hospital of University of South China and frozen in nitrogen (liquid) immediately. Extraction of total RNA was completed and then submitted to circRNA detection. This research work was done under the Ethics Committee of the Second Affiliated Hospital of University of South China approval and completed in accordance with the Declaration of Helsinki (Ethical code: LL201917). In this work, all the procedures with human participants were conducted according to the ethical standards of the medical Ethics Committee of the Second Affiliated Hospital of University of South China and the 1964 Helsinki declaration and its future amendments or comparable ethical standards. All experimental subjects provided informed consent. All procedures involving animals in this study were completed based on ethical standards for the institutional standard guidelines of the Second Affiliated Hospital of University of South China where the research was conducted.

### 2.2 Cell culturing and transfecting

Human melanoma cell lines WM‐115 and A‐375 were obtained from ATCC. Cells were incubated in a Dulbecco‐modified Eagle medium with 10% fetal bovine serum within a 5% carbon dioxide atmosphere. DNA fingerprinting was conducted to confirm cell authenticity before use. Mycoplasma infections were detected regularly.

An 873 bp cDNA fragment was cloned into PLCDH‐cir plasmid vector (Ribobio, China) constructing circITCH‐overexpressed vector. Lentivirus packages were produced by Hanbio Company. The transfects were created with Lipofectamine 3000 (Invitrogen). circITCH siRNAs, miR‐660 mimics, and inhibitors were obtained from GeneCopoeia (USA), and the siRNA sequences are shown in Table S1.

### 2.3 Quantitative real‐time PCR study

Total cellular RNA samples were extracted via TRIzol (Invitrogen). NE‐PERTM Nuclear and Cytoplasmic Extraction Reagents (Thermo Scientific) were utilized in extracting nuclear and cytoplasmic RNA fractions. SYBR Premix Ex TaqTM (Takara, Japan) plus an All‐in‐OneTM miRNA qRT‐PCR Detection Kit (GeneCopoeia) were utilized to carry out qRT‐PCR assay via Bio‐Rad IQTM5 Multicolor Real‐Time PCR Detection System (USA). qRT‐PCR primers synthesized by GeneCopoeia (Table S2) were applied in this study.

### 2.4 Cell counting kit‐8 assay

1 × 10^3^ cells were resuspended and placed into a 96‐well plate after transfection. Then the cells were kept at 37°C for 48 h before adding 10 μl CCK‐8 solution (Dojindo Laboratories, Japan). After incubation at 37°C for 2 h, the absorbance at 450 nM was recorded by a microtiter plate reader (Bio‐Tek EPOCH2, USA).

### 2.5 Formed colony analysis

All the 1 × 10^3^ cells were resuspended and seeded in 6‐well plates. After incubation for 2 weeks at 37°C, methanol was applied for colonies fixation and 0.1% crystal violet staining afterward. ImageJ software was utilized to count the colony number.

### 2.6 Transwell assay

In total, 1 × 10^4^ cells were resuspended and seeded in the top migration chambers (BD Biosciences) without FBS. Meanwhile, 10% FBS was supplemented to the lower chamber (chemoattractant). A day later, cells in the upper chambers were fixed by means of methanol then 0.1% crystal violet stained. Cell migration was visualized and counted using ImageJ software.

### 2.7 Mouse xenograft model

Animal experiments in this study were approved and executed following the procedures of the Institutional Animal Care and Use Committee of the XX. All 2 × 10^6^ cells were inoculated in the male nude mice (*n* = 5, 4 weeks old) subcutaneously. After that, 40 μl control vector or circITCH overexpression vector was intratumorally injected every 4 days. After 4 weeks, the xenograft tumors were excised under anesthesia to measure the weight of the tumor.

In the mouse lung metastasis assay, a total of 1 × 10^5^ cells were inoculated through tail veins (*n* = 5). Then, lungs were removed 8 weeks later in anesthesia. The sites of metastasis were tallied.

### 2.8 Luciferase reporter assay

A 96‐well plate was used to seed all cells (3 × 10^4^ cells in every well). The predicted circITCH and 3’‐UTR of TFCP2‐binding sites in miR‐660 had mutated. The miRNA inhibitors or mimics as well as reporter vector constructs (circITCH‐wt/mut or TFCP2 3’‐UTR‐wt/mut) were cotransfected into cells for 2 days. A dual‐luciferase reporter assay system kit (Promega) was applied to assess relative luciferase activity.

### 2.9 RNA immunoprecipitation

Transfection of cells was accomplished with MS2bs‐circITCH, MS2bs‐circITCH‐mt, and MS2bs‐Rluc. After a 48 h incubation, RIP assay was carried out using Magna RIP RNA‐Binding Protein Immunoprecipitation Kit (Millipore). The miR‐660 level was assessed after purifying RNA complexes.

The Ago2 protein assays were run using anti‐Ago2 antibody (Millipore). The relative number of circITCH, TFCP2, and miR‐660 was confirmed once RNA was purified.

### 2.10 Statistical studies

These analyses were conducted via SPSS 20.0 software. Comparative analysis among the groups was executed using *t* test. The data were offered as the mean ± SD of three independent studies unless otherwise specified. *p* < 0.05 reflected statistical significance.

## 3 RESULTS

### 3.1 circITCH is repressed in melanoma and overexpression of circITCH suppresses melanoma proliferation and metastasis in vitro

**FIGURE 1:**
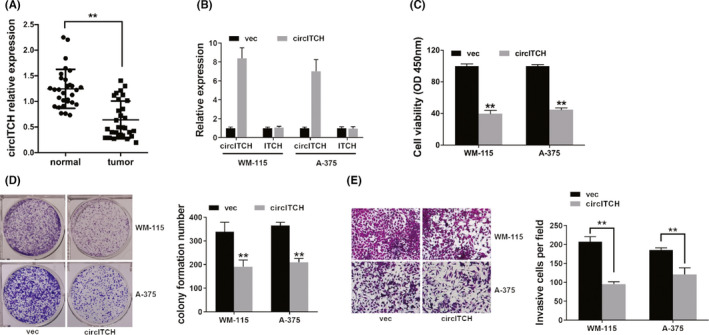
circITCH is repressed in melanoma and overexpression of circITCH lowers melanoma proliferation as well as metastasis in vitro. (A) The circITCH expression in 30 pairs of melanoma tissues and adjacent normal tissues. (B) Overexpression vector upregulated the circITCH expression. (C) CCK‐8 assay was carried out to investigate cell proliferation ability. (D) Colony formation was assayed to reveal cell colony formation capacity (left) and ImageJ software was used to quantify colony formation number (right). (E) Transwell assay was executed to assess cell metastasis (left), and ImageJ software was used to quantify invasive cell number (right). **p < 0.01

To reveal expressed circITCH in melanoma, we detected 30 melanoma tissues and adjacent normal tissues pairs. The data uncovered that circITCH was downregulated in melanoma (Figure 1A). To examine the circITCH function in melanoma, we overexpressed circITCH in melanoma (Figure 1B). We performed a CCK‐8 assay and found that overexpression of circITCH downregulated cell proliferation (Figure 1C). Moreover, overexpression of circITCH in melanoma inhibited cell colony formation ability (Figure 1D). Meanwhile, transwell analysis showed that overexpressed circITCH in melanoma suppressed metastasis (Figure 1E).

### 3.2 Overexpression of circITCH suppresses melanoma proliferation and metastasis in vivo

**FIGURE 2:**
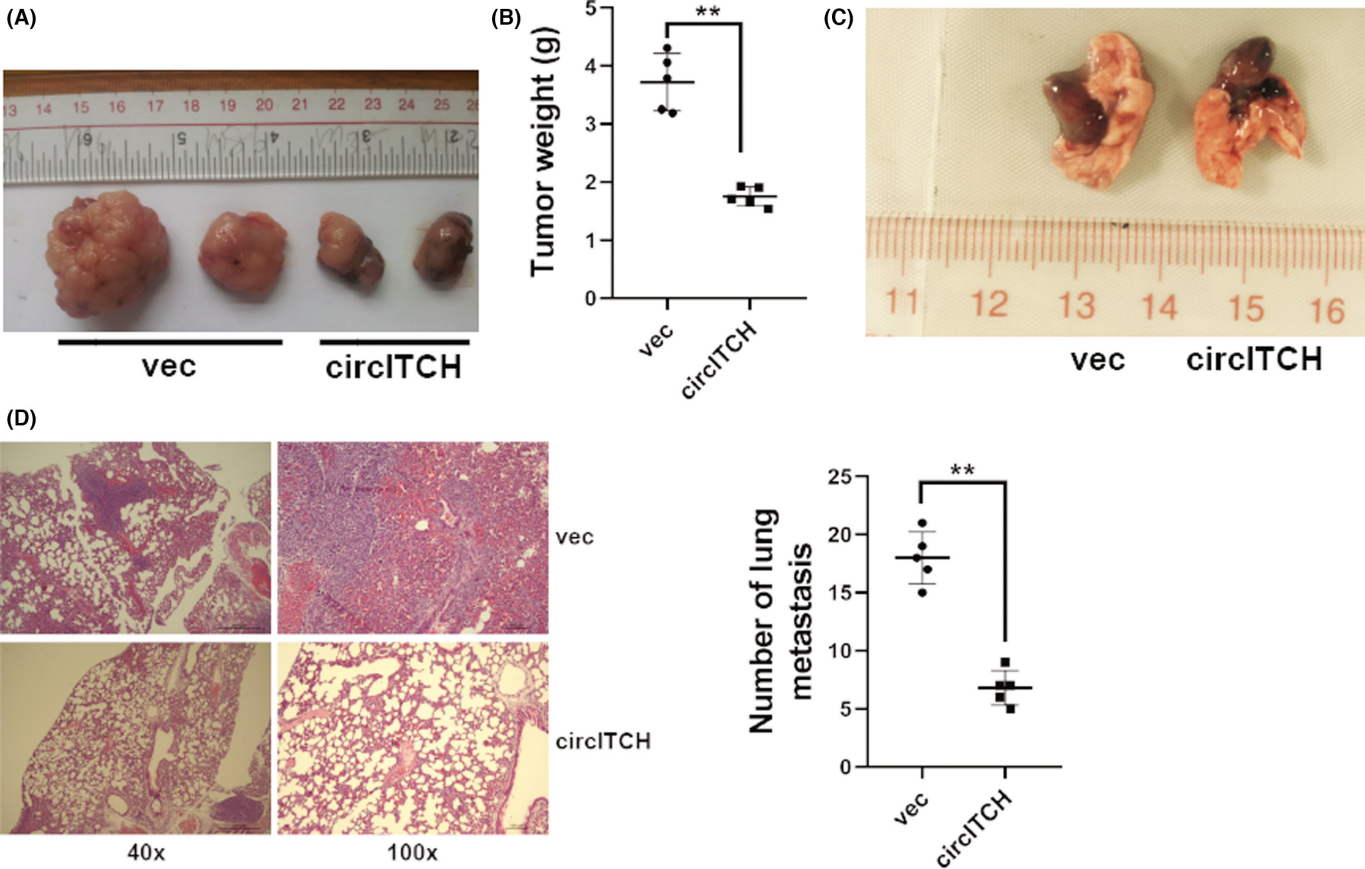
Overexpression of circITCH suppresses melanoma proliferation and metastasis in vivo. (A) Mouse xenograft prototypes evaluated circITCH function in vivo. (B) Xenograft tumor weights were shown. (C) Symbolic descriptions of lung metastatic nodes were shown. (D) HE‐stainings of metastatic sites were shown (left) and the number of metastatic sites were computed (right). **p < 0.01

We continued to explore circITCH role in melanoma in vivo using mouse xenograft models. Overexpression of circITCH suppressed tumor growth in vivo (Figure 2A,B). Moreover, overexpression of circITCH suppressed tumor metastasis in vivo (Figure 2C,D).

### 3.3 circITCH functions as a sponge for miR‐660

**FIGURE 3:**
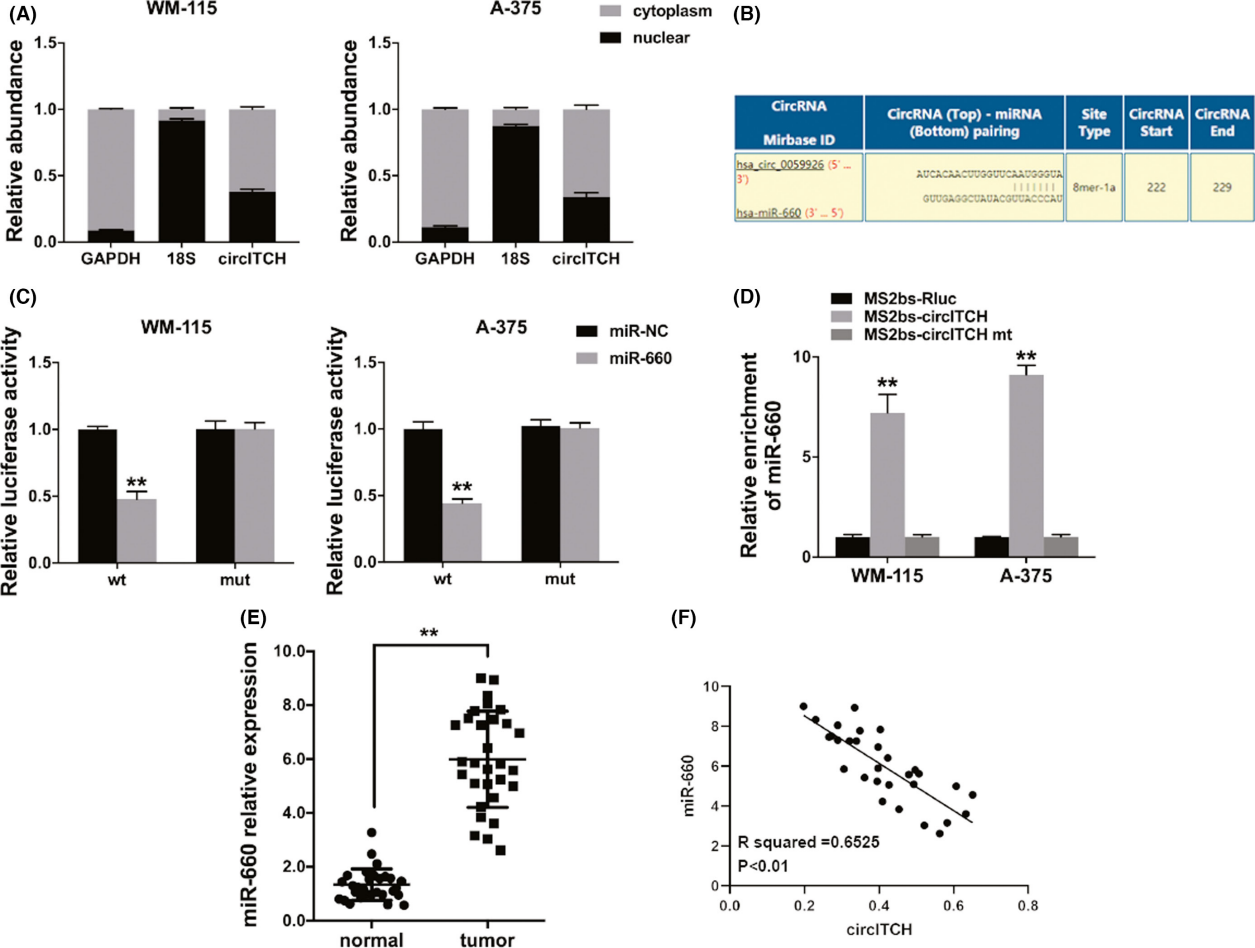
circITCH functions as miR‐660 sponge. (A) qRT‐PCR detected the cytoplasmic control (GAPDH), nuclear control (18S), and circITCH levels. (B) The predicted miR‐660‐binding sites within circITCH. (C) Luciferase assay was performed. (D) MS2 RIP assay was performed using MS2bs‐circITCH, MS2bs‐circITCH‐mt, or control. (E) qRT‐PCR detected the levels of miR‐660 30 pairs of melanoma and adjacent normal tissues. (F) Pearson correlation analysis in miR‐660 and circITCH for 30 melanoma tissues. **p < 0.01

Increasing studies reveal that circRNAs are involved in miRNA inhibition by acting as microRNA decoys.^14^ CircRNAs in the cytoplasm function as competing endogenous RNAs (ceRNAs) to sponge miRNAs as well as upregulate their target genes.^15^ Therefore, we studied the circITCH cellular location in melanoma. The results indicated that circITCH was predominantly localized in the cell cytoplasm (Figure 3A), indicating its role as an miRNA sponge. Thus, we utilized Circular RNA Interactome to explore the potential circRNA/miRNA interaction and miR‐660 was predicted (Figure 3B). Luciferase reporter assay showed reduced luciferase activity once cells were cotransfected with WT luciferase reporter and miR‐660 mimics (Figure 3C). Furthermore, to investigate direct binding among the circITCH and miR‐660, we did the RIP assay. The data disclosed predominantly enrichment of miR‐660 in MS2bs‐circITCH group (Figure 3D), indicating that circITCH openly interacts with miR‐660 and is an miR‐660 sponge. Furthermore, we examined the level of miR‐660 in 30 melanoma and adjacent normal tissue pairs which revealed that miR‐660 was upregulated in melanoma (Figure 3E). Besides, the miR‐660 expression was negatively correlated with circITCH (Figure 3F).

### 3.4 circITCH is a ceRNA regulator of TFCP2

**FIGURE 4:**
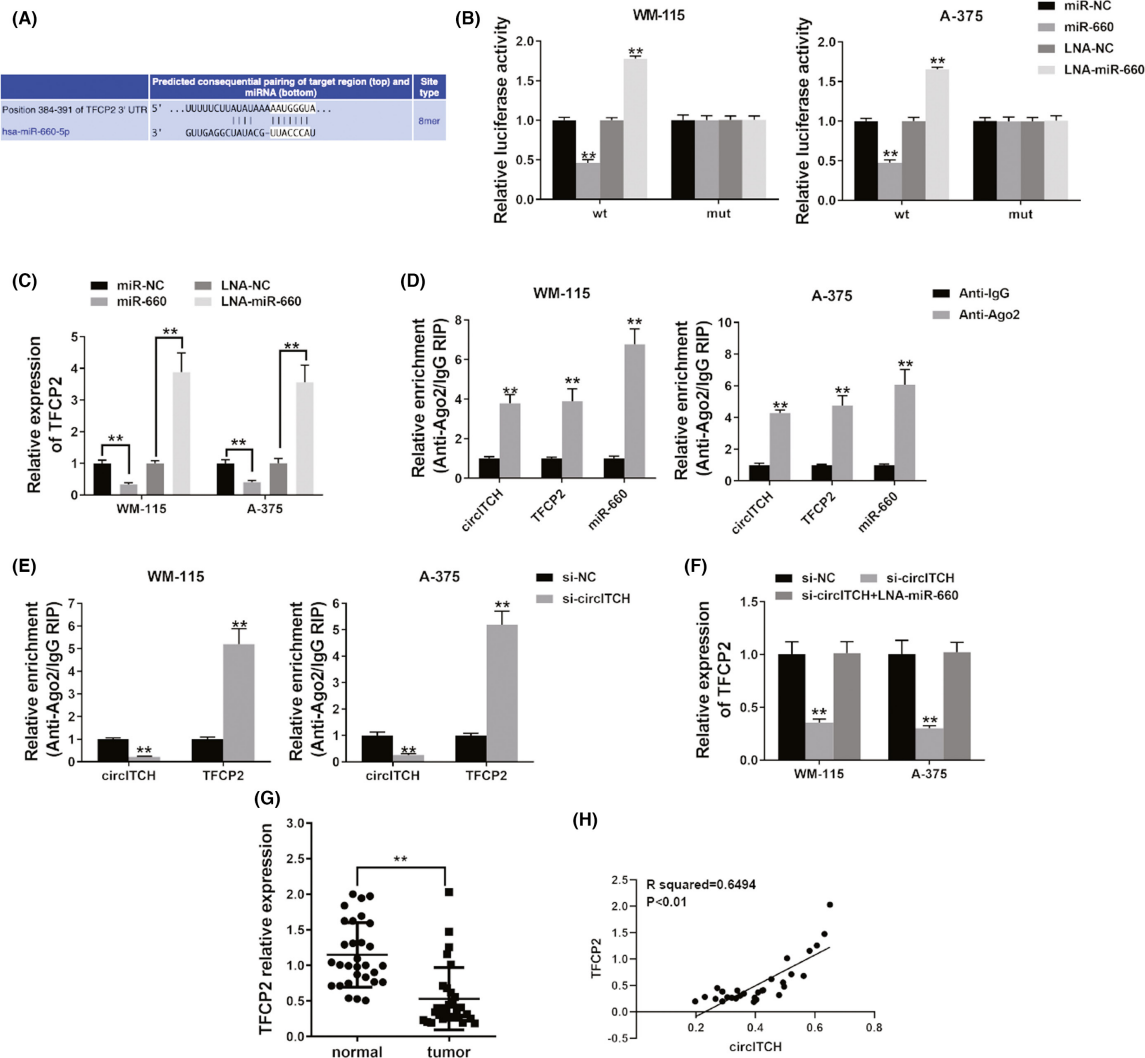
circITCH functions as ceRNA TFCP2 regulator. (A) The projected binding miR‐660 site in the 3’UTR of TFCP2. (B) Luciferase assay was performed. (C) qRT‐PCR detected TFCP2 expression after cell transfection. (D) RIP assay showed the level of circITCH, TFCP2, and miR‐660 enrichment for Ago2. (E) RIP assay on Ago2 was done after cell transfection. (F) qRT‐PCR detected TFCP2 expression after cell transfection. (G) The TFCP2 expression in 30 pairs of melanoma tissues and adjacent normal tissues. (H) Pearson correlation examination of TFCP2 and circITCH for the 30 melanoma tissues. **p < 0.01

To investigate if circITCH sponges miR‐660 to upregulate downstream target genes expression, we used TargetScan to search for possible targets of miR‐660, and TFCP2 was revealed (Figure 4A). Luciferase reporter assay showed that the luciferase ability was decreased when cotransfection of cells with miR‐660 mimics and WT luciferase reporter was completed (Figure 4B). Besides, luciferase intensity increased after cells were cotransfected with WT luciferase reporter plus miR‐660 inhibitor (Figure 4B). Moreover, we detected the expression of TFCP2 and found that miR‐660 could inhibit the expression of TFCP2, whereas the miR‐660 inhibitor upregulated the expressed TFCP2 (Figure 4C), specifying that miR‐660 was a TFCP2 regulator.

Next, we performed an RIP assay on Ago2 to investigate the underlining mechanism. We found that circITCH, TFCP2 as well as miR‐660 with predominant enrichment to Ago2 (Figure 4D), indicating that circITCH and TFCP2 could be recruited to Ago2‐related RISC and bind to miR‐660. Additionally, knockdown of circITCH reduced the enriched circITCH to Ago2, while promoting the TFCP2 enrichment to Ago2 (Figure 4E), indicating that circITCH acted as a ceRNA to compete with TFCP2 of binding miRNAs. Additionally, knocking circITCH reduced TFCP2 levels, whereas cotransfection with miR‐660 inhibitor reversed it (Figure 4F). This demonstrates that circITCH‐sponged miR‐660 controls expressing TFCP2 in melanoma. Moreover, we detected the TFCP2 expression in 30 melanoma and adjacent normal tissue pairs and found it downregulated in melanoma (Figure 4G). Expressed TFCP2 levels were found to be associated with circITCH (Figure 4H).

## 4 DISCUSSION

Melanoma is an aggressive disease that is rising in incidence. Over the past 10 years, increasing targeted agents and antibodies have prolonged the survival time of melanoma. Yet, advanced melanoma is still a life‐threatening disease.^16^ CircRNAs are part of the drivers of melanoma progression.^17^ For instance, CDR1as acts as a key player in melanoma metastasis via IGF2BP3.^18^ And circ_0020710 promotes melanoma progression and immune evasion through the miR‐370‐3p/CXCL12 axis,^19^ indicating the importance of circRNAs in melanoma.

circITCH is a key tumor suppressor in various cancers. We tested circITCH levels in melanoma tissues and found that circITCH was markedly downregulated in melanoma. Multiple studies have shown that the expression of circITCH was downregulated, and it was relatively anticancerous. It suppressed prostate cancer progress via sponging miR‐17‐5p and increasing HOXB13 expression.^20^ In glioma, circITCH exerts anti‐oncogenic effects via miR‐214 sponging and modulating the ITCH‐Wnt/β‐catenin pathway.^21^ In gastric cancer, circITCH suppresses cancer metastasis via acting as an miR‐199a‐5p sponge and increasing Klotho levels.^22^ Subsequently, CCK‐8 assay, colony formation assay, transwell analysis, and mouse xenograft models were used for circITCH overexpression in vitro and in vivo, the results revealed that overexpression of circITCH remarkably reduced the effect of circITCH on the growth, clone formation, invasion, and metastasis in melanoma, indicating that circITCH is a key tumor suppressor in melanoma and it plays a crucial part in melanoma progression.

Thus, we investigated its molecular mechanisms. CircRNAs regulate and communicate among themselves via competitive binding to share miRNAs according to the ceRNA theory.^23^, ^24^ miRNAs can be regulated by miRNA sponges, such as lncRNAs and circRNAs. In papillary thyroid carcinoma, lncRNA ASMTL‐AS1 was reported to sponge miR‐660 to upregulate FOXO1 expression, repressing glycolysis, and tumorigenesis.^25^ In melanoma, circ_0002770 is a ceRNA that stimulates cancer proliferation and invasion via targeting miR‐331‐3p.^26^ Here, we found that circITCH might sponge miR‐660 via binding with miR‐660. We found that circITCH is an miR‐660 sponge in melanoma, and regulatory relationships were verified via the dual‐luciferase reporter as well as RIP assays in cells and tissues. Relative to circITCH, miR‐660 expression was markedly increased in melanoma, further verifying the combination of the two.

Increasing studies have indicated that aberrant miRNA expressions are crucial drivers of cancer progression. miR‐660 has been identified as a tumor‐promoting miRNA. In hepatocellular carcinoma, miR‐660 stimulated the PI3K/AKT pathway that instigated EMT as well as cell cycle.^27^ Overexpression of miR‐660 promoted tumorigenesis and bone metastasis in lung cancer.^28^ miR‐660‐5p was increased in patients’ plasma and exosomes, and upregulation of miR‐660 promoted tumorigenesis by targeting KLF9 in NSCLC.^29^ In osteosarcoma, miR‐660 was significantly upregulated and served oncogenic roles by directly targeting FOXO1.^30^ In breast cancer, miR‐660 was upregulated and could regulate cancer proliferation, migration, and invasion.^31^

The functions of miR‐660 in melanoma are basically the same as the above‐reported research.

Then, we predicted and confirmed that miR‐660 binds TFCP2 3’‐UTRs, and its expression was confirmed to be markedly suppressed by miR‐660. TFCP2 plays a part in cancer development.^32^ TFCP2 could be a pro‐oncogenic feature in the liver,^33^ pancreatic,^34^ and breast cancers,^35^ and it could also be a tumor suppressor to inhibit melanoma growth.^36^ Here, we found that TFCP2 was downregulated in melanoma tissues. TFCP2 acted as a downstream miR‐660 target and could be controlled by it. And there is a positive correlation between the expression of TFCP2 and circITCH in melanoma tissues, indicating that miR‐660 and its target genes TFCP2 are both regulated by circITCH in melanoma.

The above findings imply that circITCH may play a vital role in the growth and metastasis of melanoma by circITCH/miR‐660/TFCP2 axis. We will expand the sample size of melanoma to further confirm the mechanism in the future.

## 5 CONCLUSION

Overall, our study shows that circITCH is a tumorigenesis suppressor in melanoma, mechanistically by controlling cascades of the miR‐660/TFCP2 pathway. Therefore, circITCH and its downstream pathway can effectively improve symptoms of melanoma in patients. These findings provide a theoretical basis for the development of new therapeutic strategies and have potential prognostic implications for melanoma. Further studies on circITCH may inform on the diagnostic markers for early melanoma screening.

## CONFLICT OF INTEREST

The authors have no relevant financial or nonfinancial interests to disclose.

## AUTHOR CONTRIBUTION

Conception and design: Jianfei Zhang and Bin Jiang. Administrative support: Congling Zhao. Provision of study materials or patients: Jianfei Zhang and Yanlin Cai. Collection and assembly of data: Jianfei Zhang and Shunliang Sheng. Data analysis and interpretation: Jianfei Zhang and Yanlin Cai. Manuscript writing: All authors. Final approval of manuscript: All authors. All authors read and approved the final version of the manuscript.

## ETHICS STATEMENT

All applicable international, national, and/or institutional guidelines for the care and use of animals were followed. All procedures performed in studies involving human participants were in accordance with the ethical standards of the institutional and/or national research committee and with the 1964 Helsinki declaration and its later amendments or comparable ethical standards.

## Informed consent

Informed consent was obtained from all individual participants included in this study.

## Supporting information


Table S1

Table S2

## Data Availability

Data availability statement All authors approved that all data and materials as well as software application or custom code support our published claims and comply with field standards. Data will be made available if needed.
