# Supplementary material for: circITCH suppresses cell proliferation and metastasis through miR‐660/TFCP2 pathway in melanoma
Source: Cancer Med. 2022 Mar 10;11(12):2405–13. doi: 10.1002/cam4.4627 (PMC9189461; doi:10.1002/cam4.4627)
Supplement: Supplementary file 1 — Table S1 [file CAM4-11-2405-s001.docx]

**Supplementary Table 1. The sequences of siRNAs used in this study**

| ID | Sequence |
| --- | --- |
| si-NC | UUCUCCGAACGUGUCACGUTT |
| si-circITCH #1 | CAGATCACTGAGCTGCTAACT |
| si-circITCH #2 | CTTCAGATCACTGAGCTGCTA |
| si-circITCH #3 | TTCAGATCACTGAGCTGCTAA |
