# Supplementary material for: circITCH suppresses cell proliferation and metastasis through miR‐660/TFCP2 pathway in melanoma
Source: Cancer Med. 2022 Mar 10;11(12):2405–13. doi: 10.1002/cam4.4627 (PMC9189461; doi:10.1002/cam4.4627)
Supplement: Supplementary file 2 — Table S2 [file CAM4-11-2405-s002.docx]

**Supplementary Table 2. The primer sequences for qRT-PCR used in this study**

| ID | Sequence (5’- 3’) |
| --- | --- |
| GAPDH F | TGTTCGTCATGGGTGTGAAC |
| GAPDH R | ATGGCATGGACTGTGGTCAT |
| 18S F | TTAATTCCGATAACGAACGAGA |
| 18S R | CGCTGAGCCAGTCAGTGTAG |
| TFCP2 F | GTGTTCCATGACAGAAGGCTT |
| TFCP2 R | TTATACCCACAGACATCGGGAT |
| circITCH F | GGTTCAATGGGTAGCCTCAC |
| circITCH R | GCGACTCCTCAGTCCCTAGA |
